# A Case of Thrombosed Inferior Mesenteric Artery Aneurysm Concomitant with Abdominal Aortic Aneurysm Treated by Endovascular Aneurysm Repair

**DOI:** 10.3400/avd.cr.20-00171

**Published:** 2021-06-25

**Authors:** Yoichi Yamashita, Sayako Nakagawa, Kosuke Sakamoto, Shohei Kitamoto, Taiko Horii

**Affiliations:** 1 Department of Cardiovascular Surgery, Faculty of Medicine, Kagawa University, Miki-cho, Kita-gun, Kagawa, Japan; 2 Shikoku Medical Center for Children and Adults, Zentsuji, Kagawa, Japan

**Keywords:** inferior mesenteric artery, abdominal aortic aneurysm, endovascular surgery

## Abstract

A 71-year-old man was referred to our hospital under a diagnosis of abdominal aortic aneurysm (AAA). The past history of the patient included a sigmoid colectomy at 64 years old for an ischemic colitis. The maximum diameter of AAA was still 45 mm, and the inferior mesenteric artery (IMA) was aneurysmal and was 30 mm in diameter and thrombosed. The growth rate in the diameter of IMA aneurysm was 5 mm per year for the last 3 years. The patient successfully underwent endovascular aneurysm repair (EVAR), and the postoperative course was good. At 5 years after EVAR, computed tomography revealed a decrease in the diameter of both aneurysms.

## Introduction

An aneurysm of the inferior mesenteric artery (IMA) is rare in splanchnic artery aneurysms.^1,2)^ We report a case of enlarging thrombosed IMA aneurysm (IMAA) concomitant with abdominal aortic aneurysm (AAA). Endovascular aneurysm repair (EVAR) was also effective for IMAA.

## Case Report

**Figure 1:**
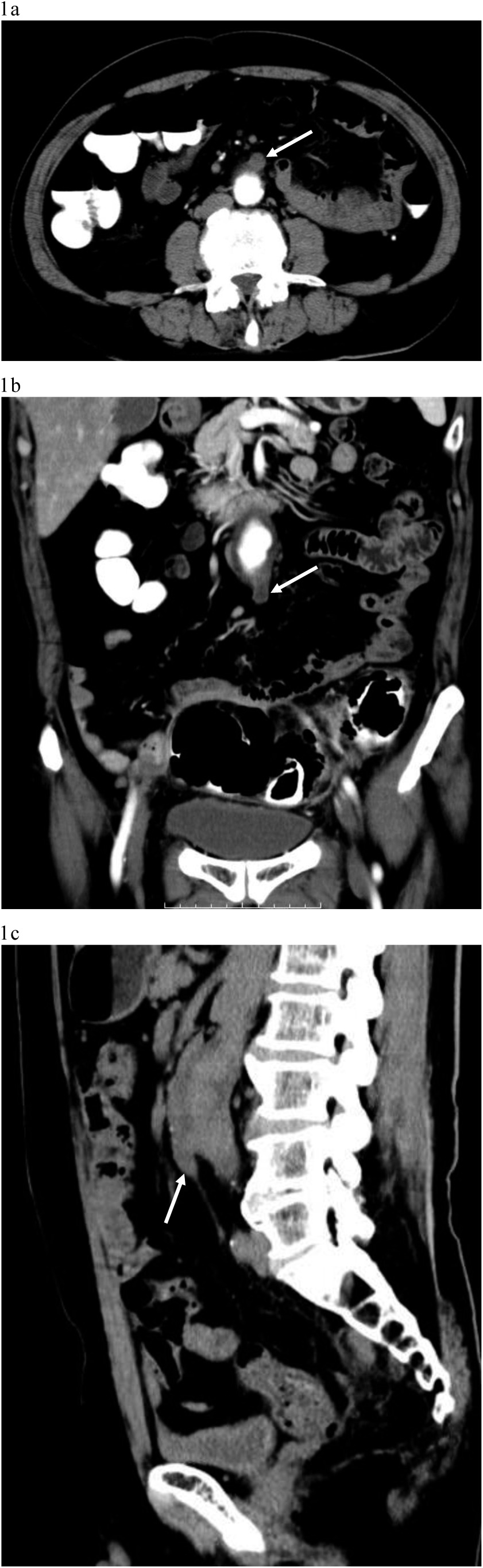
Fig. 1 Contrast-enhanced computed tomography at 7 years before endovascular aneurysm repair. (**a**), (**b**), and (**c**) show axial, coronal, and sagittal views, respectively. The inferior mesenteric artery (white arrow) is already aneurysmal and thrombosed.

**Figure 2:**
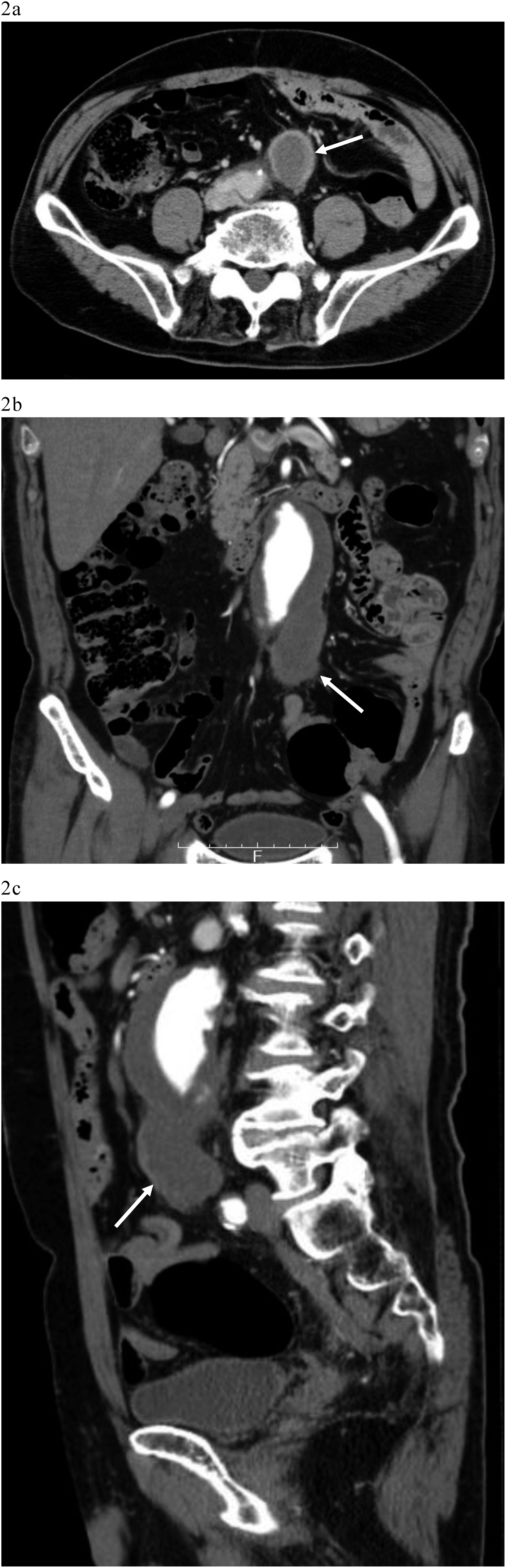
Fig. 2 Contrast-enhanced computed tomography just before endovascular aneurysm repair. (**a**), (**b**), and (**c**) show axial, coronal, and sagittal views, respectively. The inferior mesenteric artery aneurysm (white arrow) is enlarged.

A 71-year-old man was referred to our hospital under a diagnosis of AAA. The past history of the patient included diabetes mellitus, chronic type C hepatitis, and sigmoid colectomy for an ischemic colitis at 64 years old. On admission, his laboratory data was within normal range, and syphilis was negative. Computed tomography (CT) showed an infrarenal fusiform AAA and an aneurysm of the IMA that was thrombosed. CT at 7 years before, when the patient underwent sigmoid colectomy, showed both aneurysms, and the diameter of IMAA was 10 mm (**Figs. 1a**–**1c**). At the time of EVAR, the diameters of AAA and IMAA were 45 and 30 mm, respectively (**Figs. 2a**–**2c**). IMAA had enlarged 5 mm per year for the last 3 years. CT, brain magnetic resonance imaging, and coronary angiogram could not detect any other aneurysms.

The size of AAA was under the indication of the surgery; however, the size and growth rate of IMAA reached the indication of treatment. IMAA was totally thrombosed and difficult to directly approach. We decided to perform EVAR for AAA as it was also reasonable for the treatment of IMAA.

The patient successfully underwent EVAR using Endurant-II system (Medtronic Vascular Inc., Minneapolis, MN, USA) via bilateral femoral artery cutdown, and the postoperative course was uneventful. After EVAR, CT could not detect any endoleak.

**Figure 3:**
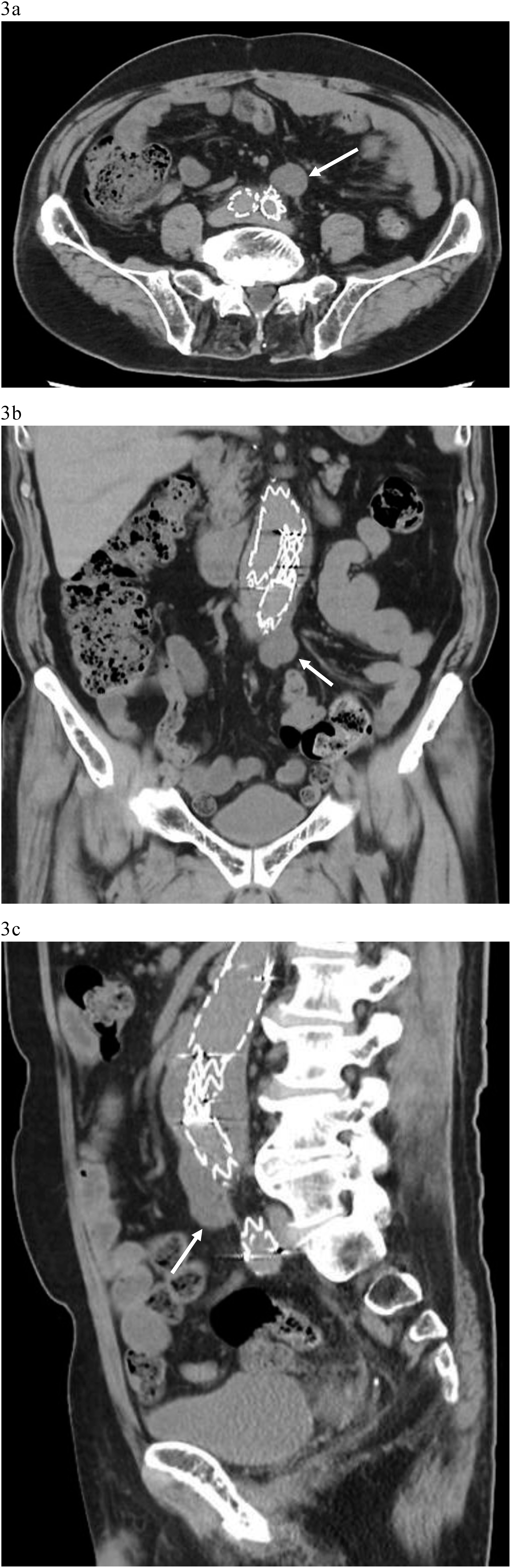
Fig. 3 Computed tomography at 5 years after endovascular aneurysm repair. (**a**), (**b**), and (**c**) show axial, coronal, and sagittal views, respectively. The size of both aneurysm is decreased.

At 5 years after EVAR, the CT showed decrease in size of both aneurysms: from 45 to 40 mm in AAA and from 30 to 19 mm in IMAA (**Figs. 3a**–**3c**).

## Discussion

**Figure 4:**
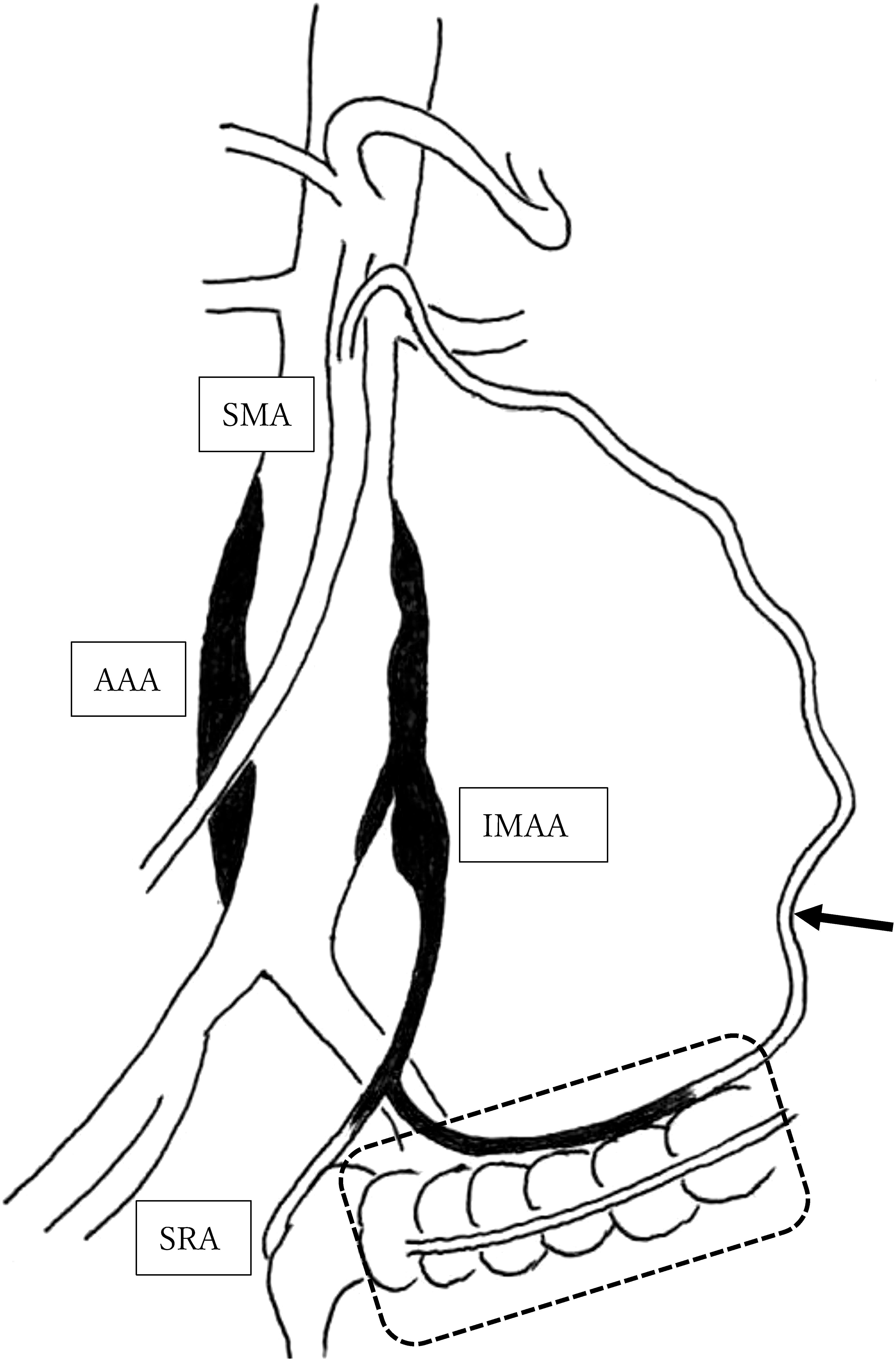
Fig. 4 Schema of interaction of the abdominal vessels and the sigmoid colon. Broken lines indicate the area of sigmoidectomy. Inferior mesenteric artery is totally thrombosed, and the lower intestine is perfused by the collateral from SMA (arrow).

AAA: abdominal aortic aneurysm; IMAA: inferior mesenteric artery aneurysm; SMA: superior mesenteric artery; SRA: superior rectal artery

IMAA is very rare, and its occurrence is reported to be 0.4%–1% of splanchnic aneurysms.^1,2)^ Nearly one-third of patients diagnosed as splanchnic artery aneurysm will have aneurysm of another area, including cerebral artery^1)^; however, in our case, no other aneurysms were detected other than AAA and IMAA. The exact rate of the concurrence of AAA and IMAA has not yet been clarified, but some describes it to be “very rare.”^3)^ The IMAA in our case was not typical as seen in previous reports.^1,3–7)^ Regardless of whether the aneurysm is fusiform or saccular, most of the splanchnic artery aneurysms have the neck and end. In our case, the proximal neck was sessile, and the wall of the aneurysm was irregular. A partially saccular aortic aneurysm with a thrombosed protrusion adjacent to the origin of IMA is one of the differential diagnosis. CT at the sigmoidectomy showed a constricted part at the base of IMA, and the distal part of the aneurysm was IMA. Therefore, we presumed that the aneurysm was IMAA. A pseudoaneurysm is another differential diagnosis.^1,7)^ There was no description of the manipulation around the IMA in the operation record of the past colectomy. The patient had no history of neither any traumatic event to abdomen nor pancreatitis. The laboratory data was within normal range, and the infection can be denial. The pathology of IMAA in our case has remained unknown because endovascular approach cannot gain any specimen of the aneurysm. The half of IMAA is atherosclerosis and is related to the superior mesenteric and celiac artery occlusion.^4–6)^ IMA was totally thrombosed, and the superior rectal artery was enhanced via unknown collateral source. The left colic artery was unclear in CT, and the proximal site of the sigmoid colon was perfused by the collateral from the superior mesenteric artery, i.e., the arc of Riolan. The celiac and superior mesenteric arteries were intact (**Fig. 4**). IMA thrombosis and decreased blood flow from the collateral source might lead to the ischemic colitis.

The timing of the treatment of IMAA is controversial. Lakin and Kashyap described ruptured cases of IMAA under 1 cm^1)^; however, Corey et al. suggested 25 mm in diameter of the asymptomatic IMAA as the indication to the treatment from their study on the natural history of the aneurysm.^2)^ Another report of IMAA suggested 2 cm in proximal and 1 cm in distal of the aneurysm as the cutoff value to treat IMAA.^5)^ The IMAA in our case was already 3 cm in diameter, and the growth rate of the aneurysm was rapid enough to be treated.

Nowadays, endovascular therapies are reported to be effective for splanchnic artery aneurysms.^6,7)^ In our case, IMAA was already thrombosed and difficult to directly approach. We expected the IMAA to shrink secondarily with the decrease of the inner pressure of AAA by EVAR; and we could achieve a good result. When IMA is patent, the proximal site should be occluded, or the chimney technique^3)^ can prevent type II endoleak from IMA,^8)^ keeping the blood flow to the intestine. The aneurysm wall was enhanced in CT just before EVAR. EVAR for AAA with the wall enhancement in CT is reported to reduce the size of AAA.^9)^ The mechanism of this phenomenon has not yet been clearly understood, and the increase of the blood flow in vasa vasorum may affect the results.

The patient has been doing well for 5 years since EVAR for AAA. A systematic review of EVAR for AAA by Wanken et al. revealed that 14% of patients that had been treated by EVAR using newer devices needed the reintervention even 7 years after EVAR.^10)^ In our case, when any type of the endoleak occurs, the increase of the inner pressure of AAA can affect IMAA. EVAR can be one of the options to treat IMAA; however, a long-term follow-up is mandatory to examine IMAA reexpansion.

## Conclusion

We report a case of thrombosed IMAA concomitant with AAA treated by EVAR.
